# The *Drosophila FM7* - *A* Strain Contains a Fixed Translocation Involving the *X* and *4* Chromosomes

**DOI:** 10.17912/micropub.biology.001664

**Published:** 2025-07-03

**Authors:** Keith Maggert, Selina Kindelay

**Affiliations:** 1 Molecular and Cellular Biology, University of Arizona, Tucson, Arizona, United States; 2 Genetics, University of Arizona, Tucson, Arizona, United States

## Abstract

We discovered that a commonly used balancer chromosome – *FM7-A, Tb* ^1^ – exists as an uncharacterized translocation between the progenitor *FM7a* chromosome and a normal chromosome *4* . Genetic and cytological data evidence show that *T* ( *1* ; *4* ) *FM7-A* translocates about 5% of chromosome *1* material to an entire chromosome *4* , thus the large “chromosome *1* ” segregant is lethal without the small “chromosome *4* ” segregant, though the latter is viable and fertile without the former. We characterize this balancer chromosome to inform *Drosophila* molecular-geneticists of the structure as it may affect their use of this balancer chromosome and the interpretations of their results.

**Figure 1. Characterization of T ( 1 ; 4 ) FM7-A , FM7a : 4 .**
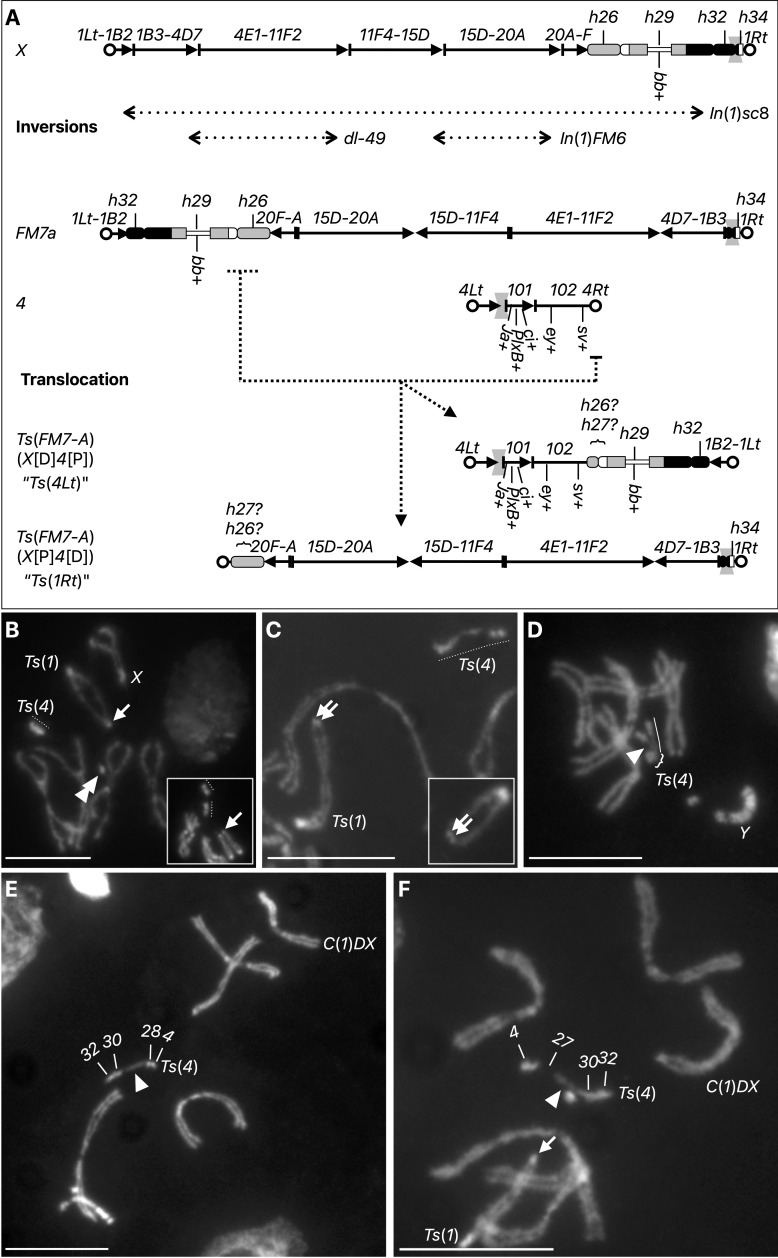
**(A) Diagrammatic representation of the translocation** , starting with the structure of a natural *X* chromosome " *X* ." Numbers and arrows indicate cytological bands defined in larval salivary glands ( *1* - *20* plus lettered and numbered subdivisions) and " *h* " numbers and rectangles indicate heterochromatic bands as defined by DAPI fluorescence in mitotic larval neuroblasts (Gatti and Pimpinelli, 1983). Three inversions occurred to convert the *X* to the *FM7a* balancer ( *In* ( *1* ) *sc8* , *dl-49* , *In* ( *1* ) *FM6* ). The translocation described in this work swaps the chromosome *1* and *4* material as indicated (dotted lines). *Lt* and circle = left telomere, *Rt* and circle = right telomere, gray spindle = centromere, *bb* + = *bobbed* (the *rDNA* locus), *sv* + = *shaven* gene, *ey* + = *eyeless* gene, *ci* + = *cubitus* *interruptus* gene, *PlxB* + = *Plexin B* gene, *Ja* + = *JYalpha* gene. Gray spindles represent centromeres. **(B) Mitotic chromosomes from *Ts* ( *1* )/ *X* ; *Ts* ( *4* )/ *4* females** . **(C) Mitotic chromosomes from *Ts* ( *1* )/ *Y* ; *Ts* ( *4* )/ *4* males. (D) Mitotic chromosomes from *C* ( *1* ) *DX* / *Y* ; *Ts* ( *4* )/ *4* females. (E) Mitotic chromosomes from *C* ( *1* ) *DX* / *Y* , *rDNA* -; *Ts* ( *4* )/ *4* females. (F) Mitotic chromosomes from *C* ( *1* ) *DX* / *Ts* ( *1* ); *Ts* ( *4* )/ *4* metafemales.** Salient chromosomes are labeled throughout, heterochromatin bands are labeled in (E) and (F); dotted line = *Ts* ( *4* ), double arrowhead = chromosome *4* , arrow = distal heterochromatic block on *Ts* ( *1* ), arrowhead = *rDNA* locus, bracket = chromosome *4* -derived sequence, line = chromosome *1* -derived sequence. Scale bars are 10 µm except for inset B which uses B scale bar at 15 µm, inset C uses C scale bar.

## Description

In the course of examining the tempo and conditions of *rDNA* magnification (Kindelay and Maggert 2024), we found that the *FM7-A* chromosome strain de-linked the complementation of a lethal null allele of the *rDNA* ( *bb* , the *bobbed* locus) from the *X* chromosome (Gatti and Pimpinelli, 1992), leading us to discover an unknown and uncharacterized chromosome translocation between chromosome *1* (the “ *FM7-A* ” *X* ) and *4* in this strain. *FM7-A* was generated by introduction of a *P* -element containing dominant *white* ^+^ and *Tubby* ^1^ alleles to the existing *X* -chromosome *FM7a* balancer chromosome (Merriam, 1969) by Ramono Lattao and colleagues (Lattao et al., 2011), and is currently available from the Bloomington *Drosophila* Stock Center (as *FM7a* , *P* { *w* ^+mC^ = *Tb* ^1^ } *FM7-A* ) (RRID:BDSC_36489). As we describe below, we believe this translocated chromosome set has breakpoints in *h26 or h27* (of the original *FM7a* ) and distal *102* (of the original chromosome *4* ) ( Figure 1A ). Any such translocation is composed of two elements which can (in principle, though not necessarily in practice) be separated through independent segregation in meiosis: here the complete genotype ( *T* ( *1* ; *4* ) *FM7-A* , *FM7a* : *4* ) has a large translocation segregant ( *Ts* ( *1* ^P^ *4* ^D^ ) *FM7-A* , henceforth in this work, “ *Ts* ( *1* )”) and a small segregant ( *Ts* ( *1* ^D^ *4* ^P^ ) *FM7-A* , henceforth “ *Ts* ( *4* )”). *Ts* ( *1* ) possesses most of the *X* euchromatin and is driven by the chromosome *X* centromere ( *4Rt* - *102* || [ *h26* - *h27* ] | *20F* - *20A* | *15D* - *20A* | *15D* - *11F4* | 4E1 - *11F2* | *4D7* - *1B3* | *h32* • *1Rt* — the brackets around *h26* - *h27* in Figure 1A indicate ambiguity in the breakpoint, and the double vertical line is the “ *FM7-A* breakpoint” defined by the translocation described herein). *Ts* ( *4* ) possesses most of chromosome *4* driven by the *4* centromere, and is appended by the tip of the *X* ( *4Lt* • *101* - *102* || [ *h27* - *h32* ] | *1B2* - *1Lt* ).

To map the breakpoints, we performed genetic crosses to ascertain if linkage of genes moved between *FM7a* and chromosome *4* in the *FM7-A* strain. We found that *y w* / *Y* ; *Ts* ( *4* ) males heterozygous for three alleles of *JY_alpha_***** ( *JY _alpha_* ^CR70483-TG4.0^ , *JY _alpha_* ^I^ , and *JY _alpha_* ^P^ ) were fertile, indicating the breakpoint does not affect, and is distal to, *JY_alpha_* , the proximal-most mutable locus on chromosome *4* (Öztürk-Çolak et al., 2024). *y w* / *Y* ; *Ts* ( *4* ) males were viable when heterozygous with *Df* ( *4* ) *M101-63a* , *Df* ( *4* ) *M101-62f* , or two alleles of *Plexin B* ( *PlexB* ^KG00878^ and *PlexB* ^MI15559-DH.GT-TG4.1^ ), indicating that the chromosome *4* breakpoint does not affect these other genes in the proximal region, and is therefore in distal *4* . Correspondingly, females and males of genotype *y w* ; *Ts* ( *4* )/ *ci gvl ey sv* were phenotypically normal for all four chromosome *4* -linked mutations, indicating that the chromosome *4* -linked breakpoint is distal to *cubitus interruptus* in cytological band *101E/F, eyeless* in proximal *102* , and *shaven* in distal 102. Finally, we were able to generate *y w* ; *Ts* ( *4* )/ *Ts* ( *4* ) females and males and maintain them as a homozygous stock, indicating that the chromosome *4* breakpoint is distal to any lethal or fertile complementation groups, including the distal-most gene identified on the right arm of chromosome *4* (the gene *Calcium-dependent secretion activator* , *Cadps* , which is homozygous lethal (Renden et al. 2001)). We conclude from these data that the chromosome *4* breakpoint is between *Cadps* and the telomere, and does not involve the movement of any genes from chromosome *4* to the *Ts* ( *1* ) element of the translocation.

*Ts* ( *4* ) dominantly ( *i.e.* , as a heterozygote) complemented strongly-expressive *bobbed* mutations induced on two separate *Y* chromosomes ( *Y* , *484* and *Y* , *183* ) in *C* ( *1* ) *DX* / *Y* ; *Ts* ( *4* )/ *4* females (Paredes and Maggert 2009), which indicates that the *X* -linked breakpoint is proximal to the *bobbed* locus in *h29* as it was inverted as part of the *h26* - *h32* block to distal *X* in the progenitor *FM7a* ( Figure 1A ).

In diploid neuroblasts, *Ts* ( *4* ) appeared much larger than a normal chromosome *4* ( Figure 1B, dotted line and double arrowhead, respectively), indicating an acquisition of a significant amount of genomic material. Correspondingly, *Ts* ( *1* ) appeared truncated relative to the regular *X* chromosome, and notably ended with a small dot of heterochromatin ( Figure 1B, arrow, see also inset). We infer this block of heterochromatin to be *h26* and perhaps up to *h29* , the location of the *rDNA* (Gatti and Pimpinelli, 1992). First, the staining with DAPI is brighter, indicating at least some heterochromatin is present. Second, the bright ends of the chromosome exhibit sister cohesion even when clearly euchromatic regions do not. Finally, the loss of the ability of the *Ts* ( *1* ) element to rescue *bobbed* alleles (while the *Ts* ( *4* ) acquired it) means that little if any of *h29* moved to chromosome *4* . We interpret the small size of this distal cohesing heterochromatin as *h26* or *h26* / *h27* . This means that the *Ts* ( *4* ) element contains *h26* / *h27* through *h32* , plus *1B2* - *1Lt* , accounting for the larger-than-normal appearance plus the new heterochromatin/euchromatin of *Ts* ( *4* ). To better resolve the heterochromatic breakpoint, we altered the *rDNA* dose to alter the condensation of the *rDNA* .

The *Ts* ( *4* ) element appears condensed in *Ts* ( *1* )/ *X* ; *Ts* ( *4* )/ *4* females ( Figure 1B ), indicating relatively inactive *rDNA* . The *rDNA* were more active in *Ts* ( *1* )/ *Y* ; *Ts* ( *4* )/ *4* males ( Figure 1C ). The pronounced activity of the *rDNA* appears as a decondensed *rDNA* chromosomal region and the chromosome *4* - and *FM7a* -derived materials are clearly discriminable (bracket and line, respectively). This is the general trend we've described before: males show more biased *X* -linked *rDNA* expression than do females (Kindelay and Maggert, 2024). In *C* ( *1* ) *DX* / *Y* ; *Ts* ( *4* )/ *4* females, which have more *Ts* ( *4* )-linked *rDNA* expression, the decondensed *rDNA* locus was evident ( Figure 1D, arrowhead). The *rDNA* was extremely decondensed when the *rDNA* were removed from the *Y* in *C* ( *1* ) *DX* / *Y* , *rDNA* ^-^ ; *Ts* ( *4* )/ *4* females ( Figure 1E, arrowhead). In *C* ( *1* ) *DX* / *Ts* ( *1* ); *Ts* ( *4* )/ *4* metafemales, though the *Ts* ( *4* ) element shows pronounced decondensation at the *rDNA* , there is no sign of decondensation on the *Ts* ( *1* ) element ( Figure 1F, arrowhead and arrow, respectively). These data indicate that the entirety of the *rDNA* , which retains normal activity and regulatory properties, were transferred to *Ts* ( *4* ). Further, the enhanced resolution clearly shows the heterochromatic bands and euchromatic *1B2* - *1Lt* sequence on the *Ts* ( *4* ) element (as indicated).

We could not generate *Ts* ( *1* )/ *Y* ; *ci gvl ey sv* males, confirming that *Ts* ( *4* ) contains genes that were originally *X* -linked that are necessary for viability. It is likely these genes correspond to cytological region *1Lt - 1B2* ( Figure 1A ), which is distalmost in *FM7a* , and contains approximately 25-30 genes (Öztürk-Çolak et al., 2024). First, the *Y* in males of this genotype supported viability of *C* ( *1* ) *DX* / *Y* females, indicating that it is not the translocation of the *bobbed* locus away from *FM7-A* that is responsible for the lethality in *Ts* ( *1* )/ *Y* ; +/+ males. Second, we reasoned that if our inability to recover *Ts* ( *1* )/ *Y* ; +/+ flies was due to loss of the now *Ts* ( *4* )-linked *X* genes, we could rescue the lethality by reintroduction of *1Lt* - *1B2* from a different source. We therefore sought to create *Ts* ( *1* )/ *Y* ; +/+ flies bearing the free duplication *Dp(1;f)1187* , which shares the *scute-8* breakpoint with *FM7a* and *FM7-A* (Sun et al. 2003). Addition of this chromosome restored viability, demonstrating that the euchromatin distal to the distal heterochromatic block on *FM7a* is missing in *Ts* ( *1* )/ *Y* ; +/+ males, and its absence is responsible for the lethality.

We cannot be certain of the location of the fusion points between chromosome *4* and the piece of *FM7a* that was translocated. The appended chromosome *FM7a* material could be in either orientation, and on either arm of chromosome 4. Also, we cannot tell whether the telomere of the *Ts* ( *1* ) element is the bona fide *1L* telomere, the *4R* telomere, another telomere, or the acquisition of telomeric activity by other DNA. These ambiguities do not affect our overall assessment of the structure of *FM7-A* , and our best guess is shown in the Figure 1A .

Our finding of a novel rearranged *X* and *4* chromosome pair in the *FM7-A* stock may be important in a number of situations. First, whole genome sequencing of mutations (or heterochromatic polymorphisms) balanced with *FM7-A* may yield erroneous results or assemblies. Second, “Basc” tests (Ashburner et al. 2011) using *FM7-A* are expected to uncover mutations on both the *X* and *4* components, altering the distribution, identity, frequency, and linkage of such alleles. Third, the use of *FM7-A* cannot be used to measure either *X* or *4* nondisjunction (or, of course, co-nondisjunction). Fourth, progeny counts of phenotypes linked to either the *X* or *4* may be altered when segregation products are present in the genotype. Fifth, and relevant to our original purpose, experiments to alter, score, or balance *bobbed* alleles are not reliable with an unmarked *bobbed* ^+^ chromosome *4* in the background. On the brighter side, our ability to generate *X* / *X* ; *Ts* ( *4* )/ *Ts* ( *4* ) flies indicates that the *Ts* ( *4* ) element may be used to increase the ploidy of the tip of the *X* chromosome, or allow the use of the element as a *bobbed* ^+^ chromosome (or a *Dp* ( *1Lt* - *1B2* )) whose segregation is independent of the *X* or *Y* .

## Methods

**General husbandry and genetic crosses**

Flies were fed standard molasses agar food and reared at 25°C. During crosses, parents were transferred on day 6 after introducing females to males and dumped from the transfer vial on day 11; virgins were collected starting day 9 and males were collected on day 16. For fly counts, data were collected on days 14 and 18. No flies were taken or counted after day 18.

For crosses testing the linkage of chromosome *4* genes, virgin females of the desired mutants were crossed to *FM7-A* males, and F1 progeny scored for the phenotype of the mutation in question. For *JY*_alpha_ , whose phenotype is sterility, males were crossed to *C* ( *1* ) *DX* virgins and presence/absence of any first instar larvae was scored after 5 days.

To determine if *Ts* ( *4* ) could be homozygosed in the absence of *Ts* ( *1* ), *ci* ^D^ virgin females were crossed to *FM7-A* males and the female and male offspring bred *inter se* . The non-Bar non-Tubby non-cubitus-interruptus progeny were then transferred and scored for three subsequent generations to assure the absence of the *ci* ^D^ and *Ts* ( *1* ) chromosomes.

To rescue *Y* -linked *bobbed* ( *rDNA* ) mutations, *FM7-A* virgin females were crossed to males bearing either *Y* , *484* or *Y* , *183* . Male offspring were then outcrossed to *C* ( *1* ) *DX* females. Rescue was determined by measuring the penetrance and expressivity of the *bobbed* alleles on the *Y* chromosomes.

For cytology, *Ts* ( *1* )/ *X* ; *Ts* ( *4* )/ *4* females and *Ts* ( *1* )/ *Y* ; *Ts* ( *4* )/ *4* males (in panels B and C, respectively) were generated by crossing *FM7-A* virgin females to *y w/Y* , *B* ^S^ males. *C* ( *1* ) *DX* / *Y* ; *Ts* ( *4* )/ *4* females and *C* ( *1* ) *DX* / *Ts* ( *1* ); *Ts* ( *4* )/ *4* metafemales (in panels D and F, respectively) were generated by crossing *C* ( *1* ) *DX* virgin females to *FM7-A* males. Generation of *C* ( *1* ) *DX* / *Y* ; *Ts* ( *4* )/ *4* females (in panel E) was done as described in the preceding paragraph, using the chromosome *Y* , *rDNA* ^SK19-bb-l^ (Kindelay and Maggert 2024).

To determine if *Ts* ( *1* ) could be homozygosed in the absence of *Ts* ( *4* ), *FM7-A* virgin females were crossed to *ci gvl ey sv* . Female progeny were each backcrossed to *ci gvl ey sv* males. cubitus-interruptus grooveless eyeless shaven Bar Tubby females and cubitus-interruptus grooveless eyeless shaven non-Bar non-Tubby males were bred *inter se* for 6 generations to look for cubitus-interruptus grooveless eyeless shaven Bar Tubby males; none were found (despite 79 cubitus-interruptus grooveless eyeless shaven Bar Tubby female, 147 cubitus-interruptus grooveless eyeless shaven non-Bar non-Tubby female, and 159 cubitus-interruptus grooveless eyeless shaven non-Bar non-Tubby male siblings).

To ascertain rescue by *Dp(1;f)1187* , cubitus-interruptus grooveless eyeless shaven Bar Tubby females from the above cross were crossed to *Dp(1;f)1187* males. Progeny were scored for the presence of non-yellow cubitus-interruptus grooveless eyeless shaven Bar Tubby males.

**Neuroblast squashes for mitotic chromosomes**

Neuroblasts were done as described (Sullivan et al. 2008). Slides were visualized on a Zeiss AxioSkop-II *mot* using 100X oil optics (Zeiss Plan-NEOFLUAR, 100X oil, NA 1.3). Images were taken with an AxioCam and post-processed for bright/contrast using GIMP 2.10.34 (rev 3) on a MacBook Pro (2023).

## Reagents

*Drosophila* strain genotypes (and their respective Bloomington Drosophila Stock Center numbers, at https://bdsc.indiana.edu, as of May 24, 2025):

*C* ( *1* ) *DX* : *C* ( *1* ) *DX* , *y* ^1^ *f* ^1^ *bb* ^0^ / *w* ^1118^ *shi* ^1^ / *Dp* ( *1* ; *Y* ) *B* ^S^ *Y* (lab strain)

*ci gvl ey sv* : *ci* ^1^ *gvl* ^1^ *ey* ^R^ *sv* ^n^ (RRID:BDSC_641)

*ci* ^D^ : *bt* ^D^ / *In* ( *4* ) *ci* ^D^ , *ci* ^D^ *pan* ^ciD^ (RRID:BDSC_638)

*Dp(1;f)1187* : *Df* ( *1* ) *sc* ^8^ , *y* ^1^ *sc* ^8^ *w* ^a^ ; *Dp* ( *1* ; *f* ) *1187* , *P* { *ry* ^+t7.2^ = *PZ* } *0801* *P* { *ry* ^+t7.2^ = *PZ* } *8-23 y* ^+^ ; *ry* ^506^ (RRID:BDSC_3940)

*FM7-A* : *FM7a* , *P* { *w* ^+mC^ = *Tb* ^1^ } *FM7-A* (RRID:BDSC_36489)

*FM7a* : *FM7a* (isolated from RRID:BDSC_3717)

*JY*_alpha_ : *y* ^1^ *w* ^*^ ; *TI* { *GFP* ^3xP3.cLa^ = *CRIMIC.TG4.0* } *JYalpha* ^CR70483-TG4.0^ / *In* ( *4* ) *ci* ^D^ , *ci* ^D^ *pan* ^ciD^ , *y* ^1^ *w* ^*^ ; *TI* { *TI* } *FRT101F* *JYalpha* ^l^ / *In* ( *4* ) *ci* ^D^ , *ci* ^D^ *pan* ^ciD^ , and *y* ^1^ *w* ^*^ ; *TI* { *TI* } *FRT101F* *JYalpha* ^P^ / *TI* { *GMR* - *HMS04515* } *Gat* ^eya^ (RRID:BDSC_97185, RRID:BDSC_605322, and RRID:BDSC_605323)

*M101* : *Df* ( *4* ) *M101-63a* / *In* ( *4* ) *ci* ^D^ , *ci* ^D^ *pan* ^ciD^ and *Df* ( *4* ) *M101-62f /Dp* ( *2* ; *4* ) *ey* ^D^ , *Ablp* ^eyD^ : *ey* ^D^ (RRID:BDSC_1082 and RRID:BDSC_9433)

*PlexB* : *y* ^1^ ; *ry* ^506^ ; *Df* ( *4* ) *M101-62f* , *P* {y ^+mDint2^ w ^BR.E.BR^ = *SUPor-P* } *PlexB* ^KG00878^ / *In* ( *4* ) *ci* ^D^ , *ci* ^D^ *pan* ^ciD^ , and *y* ^1^ *w* ^1118^ ; *Mi* { *DH.1* } *PlexB* ^MI15559-DH.GT-TG4.1^ / *In* ( *4* ) *ci* ^D^ , *ci* ^D^ *pan* ^ciD^ (RRID:BDSC_14579 and RRID:BDSC_93694)

*y w* : *y* ^1^ *w* ^67c23^ (lab strain)

*Y* , *183* : *y* ^1^ / *y* ^+^ *Y* , *P* { *w* ^-^ = *RSw* } *10B* , *rDNA* ^bb-183^ (from Paredes and Maggert 2009)

*Y* , *484* : *y* ^1^ / *y* ^+^ *Y* , *P* { *w* ^-^ = *RSw* } *10B* , *rDNA* ^bb-l-484^ (from Paredes and Maggert 2009)

*Y* , *rDNA* ^SK19-bb-l^ : *y* ^1^ *w* ^67c23^ / *Y* , *P* { *w* ^+^ *y* ^+^ = *SUPorP* } *590* , *rDNA* ^SK19-bb-l^ (from Kindelay and Maggert 2024)
